# Investigating the Expression and Function of the Glucose Transporter GLUT6 in Obesity

**DOI:** 10.3390/ijms23179798

**Published:** 2022-08-29

**Authors:** Sing-Young Chen, Ellen M. Olzomer, Martina Beretta, James Cantley, Craig S. Nunemaker, Kyle L. Hoehn, Frances L. Byrne

**Affiliations:** 1 School of Biotechnology and Biomolecular Sciences, University of New South Wales, Sydney, NSW 2052, Australia; 2 School of Medicine, University of Dundee, Dundee DD1 4HN, UK; 3 Heritage College of Osteopathic Medicine, Ohio University, Athens, OH 45701, USA

**Keywords:** obesity, mouse model, glucose transporter, islet biology

## Abstract

Obesity-related insulin resistance is a highly prevalent and growing health concern, which places stress on the pancreatic islets of Langerhans by increasing insulin secretion to lower blood glucose levels. The glucose transporters GLUT1 and GLUT3 play a key role in glucose-stimulated insulin secretion in human islets, while GLUT2 is the key isoform in rodent islets. However, it is unclear whether other glucose transporters also contribute to insulin secretion by pancreatic islets. Herein, we show that *SLC2A6* (*GLUT6*) is markedly upregulated in pancreatic islets from genetically obese leptin-mutant (*ob/ob*) and leptin receptor-mutant (*db/db*) mice, compared to lean controls. Furthermore, we observe that islet *SLC2A6* expression positively correlates with body mass index in human patients with type 2 diabetes. To investigate whether *GLUT6* plays a functional role in islets, we crossed *GLUT6* knockout mice with C57BL/6 *ob/ob* mice. Pancreatic islets isolated from *ob/ob* mice lacking *GLUT6* secreted more insulin in response to high-dose glucose, compared to *ob/ob* mice that were wild type for *GLUT6*. The loss of *GLUT6* in *ob/ob* mice had no adverse impact on body mass, body composition, or glucose tolerance at a whole-body level. This study demonstrates that *GLUT6* plays a role in pancreatic islet insulin secretion in vitro but is not a dominant glucose transporter that alters whole-body metabolic physiology in *ob/ob* mice.

## 1. Introduction

The high prevalence of obesity and associated metabolic disorders has become a major public health concern that is predicted to escalate in coming decades [1]. Obesity is closely related to type 2 diabetes, and the majority of patients with type 2 diabetes are overweight or obese [2]. Type 2 diabetes is characterized by chronic hyperglycemia due to an inability of the pancreatic beta cells to effectively compensate for insulin resistance. Pancreatic islets play a central role in the pathogenesis of diabetes, but their function and regulation remain incompletely understood. Islet biology is challenging to study in humans due to the low quantity of islets in the body, their heterogenous and disperse distribution throughout the exocrine pancreas, and a lack of effective in vivo imaging techniques [3,4]. Therefore, mouse models that mimic obesity and type 2 diabetes are essential tools for gaining a better understanding of pancreatic islet function.

*Ob/ob* mice are a common model of obesity and insulin resistance and have been widely used to study islet function [5]. These mice lack the gene for functional leptin, a satiety hormone, and quickly develop severe obesity and insulin resistance [6]. However, their phenotype is strain-dependent. *Ob/ob* mice on a C57BL/6 background exhibit remarkable islet hypertrophy and compensatory insulin secretion to achieve relatively normal blood glucose levels, while mice on a BTBR background develop severe hyperglycemia [6]. Another frequently used mouse model is the *db/db* mouse, which is commonly maintained on a BKS background and lacks the leptin receptor; this mouse also develops severe insulin resistance and hyperglycemia [6]. The *ob/ob* and *db/db* models have been important in demonstrating the efficacy of several pharmacotherapies, including exendin-4, liraglutide, and semaglutide, which have since been approved for clinical use, as well as the now-discontinued sibutramine [7,8].

The facilitative glucose transporter family (GLUTs) regulates the transport of glucose and other monosaccharides across lipid membranes in mammalian cells. There are 14 GLUT proteins in the family, several of which are exceptionally well-characterized, such as the constitutive transporters GLUT1 and GLUT2, and the insulin-sensitive GLUT4 [9]. In human islet beta cells, glucose influx is required for glucose-stimulated insulin secretion and is primarily controlled by GLUT1 and GLUT3, while GLUT2 performs the same role in rodents [10]. However, it is not known whether other GLUTs are also involved. In the publicly available Attie Lab Diabetes Database, GLUT6 mRNA is markedly upregulated in islets isolated from *ob/ob* mice compared to islets from lean littermates, on both C57BL/6 and BTBR backgrounds [11]. This suggests that GLUT6 may play a conserved role in the regulation of islet function in the obese, insulin-resistant state. However, no studies have investigated the functional role of GLUT6 in islets.

GLUT6 was previously known as GLUT9 and is most similar to GLUT8, sharing 44.8% amino acid identity [12]. GLUT6 and GLUT8 are distinct from other GLUTs, as they have a glycosylation site on loop 9 rather than loop 1 [12] and an N-terminal dileucine motif. Dileucine motifs are important for intracellular targeting [13]. GLUT6 also has the notable presence of two arginine residues in helices 7 and 8, and lacks the QLS motif in helix 7 [12].

GLUT6 is encoded for by the *SLC2A6* gene. In human tissues, *SLC2A6* mRNA is predominantly expressed in the brain, spleen, and peripheral leukocytes, but has also been detected in the pancreas and heart [12]. In mice, *Slc2a6* mRNA, but not GLUT6 protein, was detected in the brain and spleen [14]. However, GLUT6 protein was detected in mouse macrophages when they were stimulated with inflammatory molecules such as lipopolysaccharide [15]. When expressed in COS-7 cells and reconstituted into liposomes, GLUT6 exhibited significant glucose transport activity [12]. The authors predicted that GLUT6 was a high K_m_ transporter because its transport activity was detectable against background constitutive GLUT1 in 5 mM glucose, much more so than at 1 mM glucose, and its K_d_ was almost two times that of GLUT4 [12]. Furthermore, unlike GLUT4, the plasma membrane expression of GLUT6 was not sensitive to insulin, phorbol ester, or hyperosmolarity when expressed in rat adipocytes [13]. Interestingly, GLUT6 has been shown to be localized intracellularly in the lysosome in mouse macrophages [16]. Although insensitive to insulin, GLUT6 is a target gene of RelA, a transcription factor of the NFκB family [15,16]. GLUT6 may play a role in disease-related metabolism as it is upregulated in several cancers, including endometrial cancer [17]. In endometrial cancer cells, siRNA knockdown of GLUT6 has been shown to inhibit cell survival and reduced glucose uptake and glycolysis [17].

Mice lacking whole-body GLUT6 have normal body composition and glucose metabolism compared to wild-type mice when fed chow and a high-fat diet, except for a 20% decrease in adiposity in females fed a high-fat diet [14]. However, the high-fat diet-induced obesity model is distinct from the *ob/ob* model, as it exhibits only a mild disease state. In contrast, *ob/ob* mice on a C57BL/6 background demonstrate severe islet hypertrophy and extremely high plasma insulin concentrations [18]. Therefore, it is possible that the marked upregulation of GLUT6 in *ob/ob* mouse islets may have a role in islet function or hypertrophy.

To investigate the functional role of GLUT6 in islets, we created GLUT6 knockout (GLUT6KO) *ob/ob* mice and assessed body composition, glucose homeostasis, and islet function. To our knowledge, this is the first time that a glucose transporter (GLUT) has been knocked out in *ob/ob* mice. Herein, we show that *ob/ob* islets lacking GLUT6 had increased insulin secretion when stimulated with glucose but not KCl, compared to *ob/ob* islets expressing normal GLUT6. In contrast, GLUT6 deletion in islets from lean mice (*+/+* for the leptin gene) did not impact insulin secretion. Despite the changes in ex vivo glucose-stimulated insulin secretion, the GLUT6KO *ob/ob* mice had no metabolic phenotype related to body weight, body composition, or glucose tolerance.

## 2. Results

### 2.1. GLUT6 Gene Expression in Mouse and Human Islets

**Figure 1:**
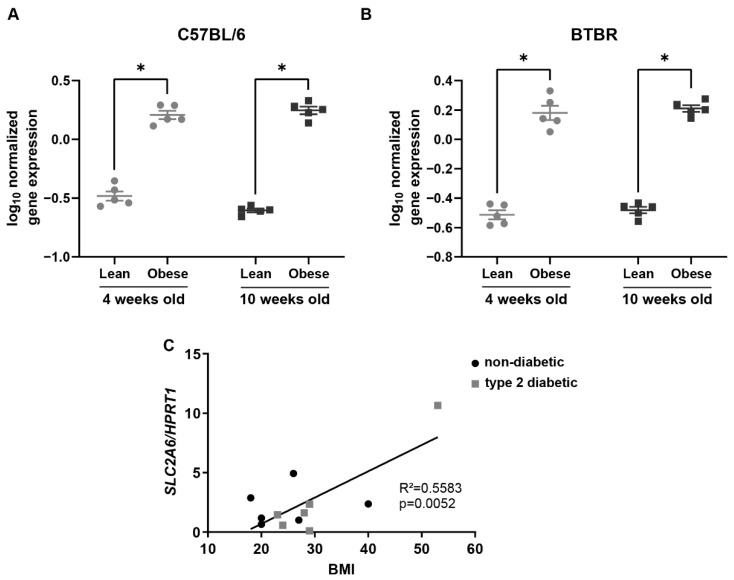
Expression of *Slc2a6* mRNA is increased in islets from obese (*ob/ob*) mice and correlates with body mass index in human islets. (**A**,**B**) *Slc2a6* expression in isolated islets obtained from lean (*ob/+* and *+/+*, grey circles) or obese (*ob/ob*, black squares) male mice (Attie Lab Diabetes Database in the form of “mlratio”, which is the log10 of the ratio of gene expression for the experimental sample to a strain-specific reference pool for either C57BL/6 (**A**) or BTBR (**B**) mice). (**C**) Human islet *SLC2A6* expression positively correlated with body mass index (BMI). Islets were isolated from non-diabetic humans (black circles) and humans with type 2 diabetes (grey squares). * indicates *p* < 0.05 by two-way ANOVA. n = 5 mice per group (**A**,**B**), n = 12 human samples (**C**).

Data mining the publicly available Attie Lab Diabetes Database [11] revealed that *Slc2a6* is highly overexpressed in the pancreatic islets of obese mice. Specifically, islets from obese mice that lack the functional leptin gene (*ob/ob*) expressed significantly higher *Slc2a6* compared with lean mice (*ob*/+ and +/+ genotypes) at both four and ten weeks of age, on both C57BL/6 and BTBR backgrounds (Figure 1A,B). To investigate whether *GLUT6* expression was also upregulated in obese and insulin-resistant human islets, we analyzed *SLC2A6* mRNA levels in pancreatic islets from a cohort of human patients with and without type 2 diabetes with a body mass index (BMI) between 18 and 53. Donor information is as previously published [19] and given in Table S1. These data showed that human islet *SLC2A6* mRNA was significantly correlated with body mass index (*p* = 0.005) (Figure 1C).

**Figure 2:**
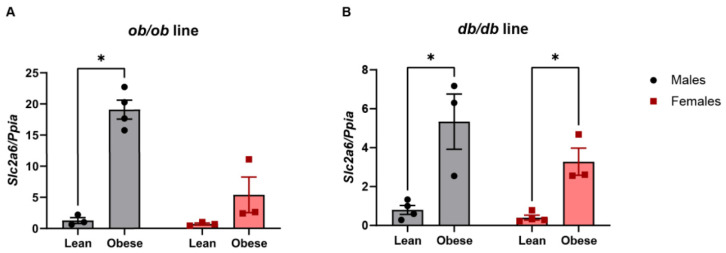
*Slc2a6* mRNA expression was increased in islets obtained from obese *ob/ob* and *db/db* mice, compared to lean control mice. *Slc2a6* expression data from isolated mouse islets of *ob/ob* (**A**) and *db/db* (**B**) mice, including males (black circles) and females (red squares). * indicates *p* < 0.05 by two-way ANOVA. n = 3–4 mice per group.

We then examined the expression of *Slc2a6* mRNA in islets from genetically obese mice in our laboratory. To do this, islets were isolated from genetically obese and lean mice of both the *ob/ob* line and the *db/db* line. Mice with two copies of the non-functional mutant leptin gene (*ob/ob*) or mutant leptin receptor gene (*db/db*) develop hyperphagia and obesity, despite consuming a normal chow diet. Only one functional copy of the leptin gene or leptin receptor gene is needed to maintain normal physiology, so the controls included littermate heterozygous (e.g., *ob*/+ or *db*/+) and wild-type (+/+) mice. The expression of *Slc2a6* was measured by qPCR and confirmed to be highly upregulated in obese *ob/ob* and *db/db* islets from male mice, with relative mRNA levels that were 6.6–12 times higher compared to the lean controls (Figure 2). *Slc2a6* mRNA was also increased in islets from obese female mice, but to a lesser extent than in the males (Figure 2). However, GLUT6 protein expression was too low for detection in islets from male mice (Figure S4). *GLUT6* knockout was not compensated for by increased GLUT2 expression (Figures S5B and S6). As the upregulation of *Slc2a6* was more evident in males, only male mice were studied in subsequent experiments.

### 2.2. Effect of GLUT6 Knockout on Insulin Secretion in Isolated ob/ob Mouse Islets

**Figure 3:**
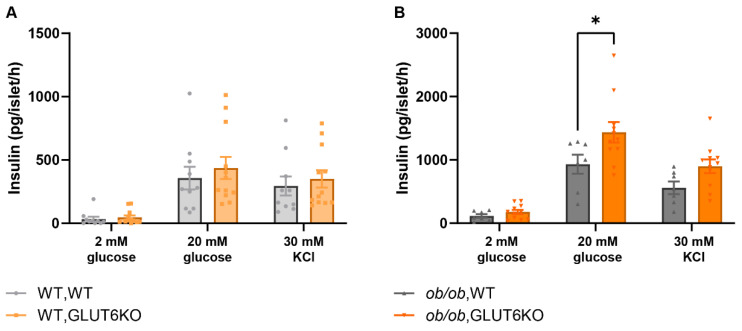
Ex vivo glucose-stimulated insulin secretion was increased in *GLUT6*-deficient *ob/ob* islets. Glucose- and potassium-stimulated insulin secretion was measured in isolated, cultured islets from wild-type (WT, **A**) or leptin-deficient *ob/ob* male mice (*Lep* genotype, **B**) with wild type (WT) or knockout (KO) of *Slc2a6*. Genotypes are WT, WT (light grey circles), WT, GLUT6KO (light orange squares), *ob/ob*, WT (dark grey triangles) and *ob/ob,* GLUT6KO (dark orange triangles). * indicates *p* < 0.05 by two-way ANOVA. n = 7–12 mice per group.

To determine whether *GLUT6* played a functional role in mouse islet glucose metabolism, we crossed the *ob/ob* line with *GLUT6*/*Slc2a6* knockout (GLUT6KO) mice. We have previously reported that *GLUT6* is successfully knocked out in this line [14], and confirmed this by qPCR analysis of isolated islets (Figure S5A). Glucose-stimulated insulin secretion (GSIS) was determined in islets from male mice of the following genotypes: wild type (WT, WT), obese (*ob/ob*, WT), *Slc2a6* knockout (WT, KO), and obese with *Slc2a6* knockout (*ob/ob*, KO) (Figure 3). The GSIS assay is an ex vivo measurement of the islets’ insulin secretory function [20]. Pancreatic islets were isolated from 16-week-old mice (of the previously mentioned genotypes) and incubated in low (2 mM) or high (20 mM) glucose, or 30 mM KCl, to determine glucose- and potassium-stimulated insulin secretion, respectively. Potassium bypasses the glucose-sensing pathway to directly cause the depolarization of beta cells, thus inducing insulin release.

Knockout of *GLUT6* did not have any impact on glucose or potassium-stimulated insulin secretion in mice with normal leptin expression (Figure 3A). However, when compared to *ob/ob* WT mice, *ob/ob* mice lacking *GLUT6* showed significantly greater glucose-stimulated insulin secretion and a trend towards increased KCl-stimulated insulin secretion (Figure 3B). These data indicate that the loss of *GLUT6* increased islet secretion of insulin in response to high glucose concentrations only in obese *ob/ob* mice.

### 2.3. Effect of GLUT6 Knockout on Whole-Body Metabolic Phenotypes in ob/ob Mice

**Figure 4:**
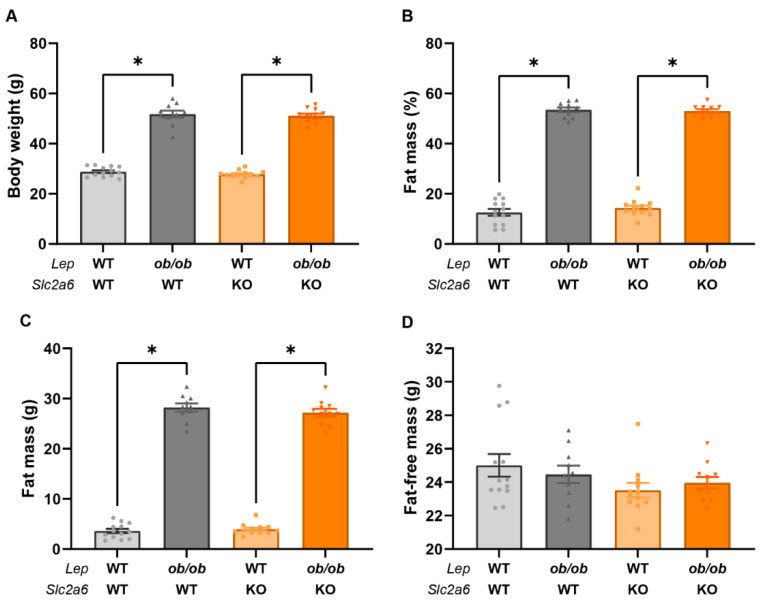
Body weight and composition were altered by leptin (*Lep*) but not by the *Slc2a6* genotype at 16 weeks of age in male mice. Body weight (**A**), fat mass as percentage of total body mass (**B**), fat mass as a raw value (**C**), and fat-free mass (**D**) were measured when mice were 16 weeks of age for genotypes WT, WT (light grey circles), *ob/ob*, WT (dark grey triangles), WT, KO (light orange squares) and *ob/ob,* KO (dark orange triangles). * indicates *p* < 0.05 by one-way ANOVA. n = 10–13 mice per group.

To investigate whether the loss of *GLUT6* impacted metabolic physiology and glucose metabolism in genetically obese *ob/ob* mice, mouse body weights, body composition, and glucose tolerance were examined at 16 weeks of age (Figure 4). At 16 weeks of age, leptin-deficient *ob/ob* mice had significantly higher body weight and fat mass compared to their corresponding lean controls (Figure 4A–C), while fat-free mass was not significantly changed (Figure 4D). However, the loss of *GLUT6* did not impact body weight or composition (Figure 4).

**Figure 5:**
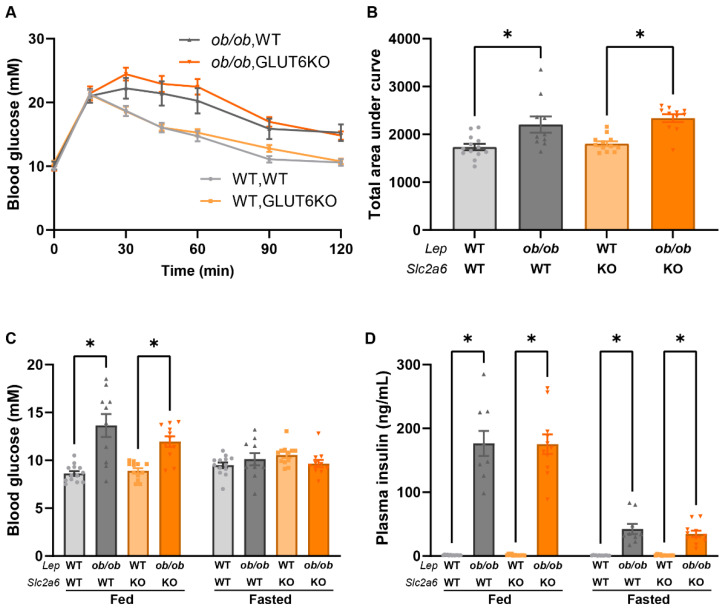
*GLUT6* knockout has no effect on glucose tolerance in mice expressing either wild-type or mutant leptin. Male mice at 16 weeks of age were assessed for glucose tolerance (**A**,**B**), blood glucose (**C**), and plasma insulin (**D**). Genotypes are WT, WT (light grey circles), *ob/ob*, WT (dark grey triangles), WT, KO (light orange squares) and *ob/ob,* KO (dark orange triangles) * indicates *p* < 0.05 by one-way ANOVA. n = 10–13 mice per group.

Similarly, glucose tolerance was impaired in *ob/ob* mice, but *GLUT6* knockout had no effect when compared to control mice expressing wild-type *GLUT6* (Figure 5A,B). Blood glucose concentrations were elevated in fed, but not fasted, *ob/ob* mice, compared to lean controls (Figure 5C). Plasma insulin levels were also increased in *ob/ob* mice in both the fed and fasted states, but the *Slc2a6* genotype did not affect plasma insulin concentrations (Figure 5D). Similar results were observed at 10 weeks of age, except that fasted blood glucose was significantly increased in obese mice that were WT for *Slc2a6*, but not in *GLUT6* knockout mice (Figures S2 and S3).

## 3. Discussion

Based on the observations that *Slc2a6* is highly upregulated in islets from obese mice and *SLC2A6* expression in islets correlates with BMI in diabetic humans, we speculated that *GLUT6* may play a role in islet function in the obese state. We previously did not observe any major effects of whole-body *GLUT6* knockout in mice subjected to diet-induced obesity [14]. In the current study, we characterized the effects of a global *GLUT6* knockout in a more severe model of obesity and beta cell compensation: the leptin-deficient *ob/ob* mice.

Under in vitro conditions, pancreatic islets isolated from *ob/ob* mice lacking *GLUT6* secreted more insulin in response to high glucose (20 mM) compared to *ob/ob* mice that expressed *GLUT6*. It is well established that islet immune cell infiltration is increased in islets from human patients with type 2 diabetes, as well as in diabetic mice, in several different models of obesity and diabetes [21]. This inflammation impairs beta cell function and contributes to blood glucose dysregulation. Since *GLUT6* is known to be markedly upregulated in response to inflammatory stimuli [14,15] and may mediate some downstream effects, it is plausible that the absence of *GLUT6* may have decreased these potential pro-inflammatory responses, thus minimizing stress on the beta cells. However, while the high concentration of glucose used in the GSIS assay is ideal for evaluating islet function, it is important to note that this condition may not be physiologically relevant. Therefore, in vivo analyses were performed to investigate the changes in physiology among mice that were lean or obese, with and without GLUT6. These whole-body studies showed that there was no observable phenotypic difference between these animals in terms of body mass, body composition, or glucose tolerance. Hence, the in vitro observation of increased GSIS in *GLUT6*KO obese islets did not translate to altered whole-body in vivo physiology in this model. One explanation for this finding is that islet *GLUT6* confers a restraining effect on insulin secretion at hyperglycemic concentrations of glucose, which are not present in the *ob/ob* model, but may be relevant during obese type 2 diabetes. Future studies in *ob/ob* mice on a BTBR background or *db/db* mice on a BKS background, which demonstrate marked hyperglycemia even in a fasted state [6], may show a difference between WT and GLUT6KO mice. However, it is also possible that *GLUT6* is a marker of the obese islet, rather than playing a key causal or reactive role. *GLUT6* may also play a role independent of the functions evaluated in our study.

Insulin-secreting beta cells are the most abundant cell type in the pancreatic islet and comprise approximately 60–80% of the mouse islet [22]. The second most common cell type is the glucagon-secreting alpha cell. However, there are also many less abundant cell types found in islets, including other endocrine cell types such as delta cells, epsilon cells, and pancreatic polypeptide (gamma) cells, as well as immune cells. Given that *Slc2a6* is known to be expressed in leukocytes [12] and is upregulated in response to inflammatory stimuli [15,16], it is plausible that the overexpression of *Slc2a6* in obese islets is primarily due to the upregulation of *Slc2a6* expression in islet immune cells and/or the higher immune cell content in obese or diabetic islets [21]. A single-cell RNA sequencing study of human islet cells revealed that *SLC2A6* mRNA transcripts ranked within the top 1140 transcripts in mast cells and MHCII-positive cells. In contrast, *SLC2A6* transcripts ranked below the top 4000 and 5000 transcripts in islet endocrine and exocrine cells, respectively [23]. In mouse islets, single-cell RNA sequencing showed that *Slc2a6* was enriched in a cluster of cells primarily comprised of cells exposed to the pro-inflammatory cytokine IL-1β and highly enriched for *Nos2* mRNA [24]. *Nos2* is an inflammatory gene induced by NF-κB downstream of IL-1β stimulation. Since IL-1β levels are associated with obesity in humans and mice [25,26] and we have previously shown that *GLUT6* expression is regulated by NF-κB [27], it is also possible that *GLUT6* expression may be elevated in specific β-cells clusters within islets. Future studies using oligonucleotide in situ hybridization or cell sorting techniques may shed further light on the cell type in which *GLUT6* is expressed in islets from obese mice.

This study has a number of limitations which should be acknowledged. The mouse model investigated involved the whole-body knockout of *GLUT6*, rather than islet-specific *GLUT6* deletion. However, due to the diversity of cell types in the islet and their complex lineages, it would have been impractical to develop a cell-type-specific *GLUT6* knockout mouse for this study. Moreover, as no change in phenotype was observed in this whole-body *GLUT6* knockout model, it is unlikely that a tissue-specific knockout would yield significant further insight. Another limitation is that the GSIS results for islets from these mice were normalized to islet number. Knockout of *GLUT6* did not change islet size; however, since *ob/ob* and WT islets typically differ in size and therefore beta cell number [28,29], comparisons between the *ob/ob* and WT genotypes should be made with caution. Unfortunately, we were unable to normalize the data to DNA content, which is standard practice in the field.

This study was also limited by the small sample size (n = 12) in the human islet study, due to the scarcity of available islets from human donors. Future studies with larger sample sizes may provide further insights into the relevance of these findings to the human disease state.

In conclusion, despite a marked increase in *Slc2a6* mRNA expression in obese mouse and human islets, whole-body *GLUT6* knockout in leptin-deficient mice did not produce an observable change in body weight, body composition, or glucose tolerance. However, *ob/ob* GLUT6KO islets showed enhanced GSIS ex vivo, suggesting that *GLUT6* may restrict insulin secretion in response to a hyperglycemic stimulus. Further investigation is required to elucidate the precise role of *GLUT6* in pancreatic islet function.

## 4. Materials and Methods

### 4.1. Mouse Colonies

The *ob/ob* mouse line (B6.Cg-Lep^ob/Aus^) was established using breeders provided by the laboratory of Professor Greg Cooney from the University of Sydney, Australia. Heterozygous mice (*ob/+*) from the *ob/ob* line were crossed with homozygous GLUT6KO mice (C57BL/6-Slc2a6^em1Ausb/Ausb^) from our GLUT6KO line, as has been previously described and validated [14]. Offspring genotypes were determined by PCR amplification and gel electrophoresis. The genotype for *Slc2a6*, the gene encoding for GLUT6, was determined according to [14]. Genotyping for the *Lep* gene was performed as previously described [30].

*Db/db* mice (BKS.Cg-Dock7^m^ +/+ Lepr^db^/J^Ausb^) were obtained from breeders provided by Professor Kerry-Anne Rye’s laboratory at the University of New South Wales, Australia. Offspring genotypes were determined by PCR amplification and gel electrophoresis according to [31].

### 4.2. Mouse Studies

All mouse experiments were approved by the University of New South Wales (UNSW) Animal Care and Ethics Committee (approval number 20/67A). Mouse breeding was conducted at Australian BioResources (Moss Vale, NSW, Australia) before the mice were issued to the Wallace Wurth animal facility at UNSW. Mice were housed at 22 °C in a 12 h light/dark cycle and provided with ad libitum access to water and a standard chow diet (Gordons Specialty Feeds, Yanderra, NSW, Australia). Mice were monitored according to ethical guidelines. Body composition was measured by quantitative magnetic resonance imaging using an EchoMRI Body Composition Analyzer (EchoMRI^TM^). At the experimental endpoint, mice were culled by cervical dislocation.

### 4.3. Glucose Tolerance Tests

Intraperitoneal glucose tolerance tests were conducted following a 6 h daytime fast. Mice were administered 33.3% D-glucose in saline by intraperitoneal injection at a dose of 2 g/kg lean mass. Blood glucose was measured at the specified time points using an Accu-Check Performa II glucometer (Roche, North Ryde, NSW, Australia).

### 4.4. Plasma Insulin Measurements

Approximately 40 µL of blood from the tail tip was collected in heparinized capillary tubes (Sarstedt 16.443, Mawson Lakes, SA, Australia) in the random-fed state and after a 6 h daytime fast. Samples were centrifuged at 2000× *g* and 4 °C for 10 min to extract the plasma, which was stored at −80 °C. Plasma insulin was measured using the Crystal Chem Ultra-Sensitive Mouse Insulin ELISA Kit (Crystal Chem 90080, Elk Grove Village, IL, USA) according to the manufacturer’s instructions, except that samples were incubated overnight at 4 °C.

### 4.5. Mouse Islet Isolation

Islet isolation and culture was based on our published protocol [20], involving collagenase digestion of the pancreas and the Ficoll gradient separation of islets. Detailed methods are available in the Supplementary Materials. Islets were cultured overnight in a RPMI-1640 medium (Thermofisher 11875-093, Waltham, MA, USA) supplemented with 10% fetal bovine serum (FBS), 15 mM HEPES, 100 U/mL penicillin, and 0.1 mg/mL streptomycin at 37 °C and 5% CO_2_ before GSIS assays, or washed in PBS and stored at −80 °C prior to RNA extraction.

### 4.6. Ex Vivo Glucose-Stimulated Insulin Secretion

Insulin secretion assays were conducted as previously described [20]. Mouse islets were incubated for 1 h at 37 °C in HEPES-buffered Kreb’s Ringer Buffer (KRBH) containing 0.1% bovine serum albumin (BSA) and 2 mM D-glucose. Batches of five size-matched islets were handpicked and stimulated by incubation at 37 °C in KRBH containing glucose or KCl for 1 h. The reaction was terminated by placing the samples on ice and centrifuging at 1000 rpm for 1 min to pellet the islets. Supernatants containing secreted insulin were collected for analysis using an in-house ELISA assay, which is described in the Supplementary materials (validation of standards shown in Figure S1).

### 4.7. Human Islet RNA Extraction and Quantitative Real-Time PCR (qPCR)

The source of the human islet samples from patients with or without type 2 diabetes used herein has been described previously [19]. Ethics approval was obtained from the University of Virginia Institutional Review Board (protocol #14904). Informed consent was obtained from the donors and their families before organ collection by the local organ procurement agency. RNA isolation and qPCR were performed according to [19]. In brief, RNA was extracted using an AllPrep DNA/RNA/Protein mini kit (Qiagen, Hilden, Germany). cDNA was obtained by reverse transcription using 0.25–1 µg RNA with a Life Technologies High-Capacity cDNA kit (Thermofisher 4368814, Waltham, MA, USA). Quantitative real-time PCR (qPCR) was conducted using the following primer sequences for *SLC2A6* (forward primer: GCC CGG ACT ACG ACA CCT, reverse primer: AGC TGA AAT TGC CGA GCA C) and the housekeeping gene *HPRT1* (forward primer: ATG GAC AGG ACT GAA CGTCT, reverse primer: TCC AGC AGG TCA GCA AAG AA). iQ SYBR green SuperMix (Bio-Rad, Hercules, CA, USA) was used to detect amplification products on an iCycler (MyiQ Optical Module) Bio-Rad System. Quantification was performed using the Pfaffl method [32].

### 4.8. Mouse Islet RNA Extraction and qPCR

RNA extraction was performed on isolated mouse islets using a TRI reagent according to the manufacturer’s protocol (Sigma T9424, Castle Hill, NSW, Australia). cDNA synthesis was performed using 225 to 350 ng RNA and an iScript cDNA synthesis kit (Bio-Rad) according to the manufacturer’s protocol.

Quantitative real-time PCR (qPCR) was conducted using the following primer sequences for *Slc2a6* (forward primer: GCG ACT CCT GGA GAG AGA GA, reverse primer: CAG GAT GCC TGG ATT TTG TC), *Slc2a2* (forward primer: TGT GCT GCT GGA TAA ATT CGC CTG, reverse primer: AAC CAT GAA CCA AGG GAT TGG ACC) and the housekeeping gene *Ppia* (forward primer: CGA TGA CGA GCC CTT GG, reverse primer: TCT GCT GTC TTT GGA ACT TTG TC). PCR products were amplified in the following (15 µL) reaction: 700 nM primers, 7.5 µL iTaq Universal SYBR Green Supermix (Bio-Rad), 3.5 µL nuclease-free water, and 2 µL cDNA, using an Applied Biosystems ViiA7 Real-Time PCR System (Thermofisher, Waltham, MA, USA). PCR cycling conditions were as follows: 95 °C (20 s), 40 cycles (95 °C (1 s) and 60 °C (20 s)) and melt curve stage (95 °C (15 s), 60 °C (1 min), and 95 °C (15 s)). qPCR analysis was performed using the Pfaffl method [32].

### 4.9. Statistical Analysis

All statistical analyses were conducted using Graphpad Prism 9.3.1 (San Diego, CA, USA). Data show mean ± SEM, and data points indicate biological replicates unless otherwise specified. A p-value of less than 0.05 was considered statistically significant.

For gene expression data with more than two groups, significance was determined by two-way ANOVA with Sidak’s correction. When comparing only two groups, the Student’s T-test was performed. Pearson’s correlation was conducted on human *SLC2A6* expression and BMI data. For body weight, body composition, and glucose tolerance data that were normally distributed with standard deviations that were not significantly different according to the Brown–Forsythe test, significance was assessed by one-way ANOVA with Sidak’s correction. For data that failed the Brown–Forsythe test, Welch’s one-way ANOVA with Dunnett’s correction was performed instead. The Kruskal–Wallis test with Dunn’s correction was utilized for non-parametric data. For fed and fasted glucose and insulin data, significance was assessed by two-way ANOVA with multiple comparisons using Graphpad Prism QuickCalcs, which performs Bonferroni’s correction and allows for the manual selection of relevant comparisons to avoid inappropriate comparison of groups where both *Lep* and *Slc2a6* genotypes were different.

For GSIS data, data points indicate biological replicates, which are the average of 4 technical replicate wells per mouse. Any outlier wells were removed using Grubbs’ test [33]. Significance was assessed by two-way ANOVA with Sidak’s multiple comparisons test.

## Data Availability

Data are available upon reasonable request to the corresponding author.

## Supplementary Materials

The following supporting information can be downloaded at: https://www.mdpi.com/article/10.3390/ijms23179798/s1.

## References

[1] Malik V.S., Willet W.C., Hu F.B. (2020). Nearly a decade on—Trends, risk factors and policy implications in global obesity. Nat. Rev. Endocrinol..

[2] Leitner D.R., Frühbeck G., Yumuk V., Schindler K., Micic D., Woodward E., Toplak H. (2017). Obesity and Type 2 Diabetes: Two Diseases with a Need for Combined Treatment Strategies—EASO Can Lead the Way. Obes. Facts.

[3] Pandiri A.R. (2014). Overview of exocrine pancreatic pathobiology. Toxicol. Pathol..

[4] Wei W., Ehlerding E.B., Lan X., Luo Q.-Y., Cai W. (2019). Molecular imaging of β-cells: Diabetes and beyond. Adv. Drug Deliv. Rev..

[5] Lindström P., Islam M.S. (2010). β-Cell Function in Obese-Hyperglycemic Mice [ob/ob Mice]. The Islets of Langerhans.

[6] Clee S.M., Attie A.D. (2007). The Genetic Landscape of Type 2 Diabetes in Mice. Endocr. Rev..

[7] Nilsson C., Raun K., Yan F.-f., Larsen M.O., Tang-Christensen M. (2012). Laboratory animals as surrogate models of human obesity. Acta Pharmacol. Sin..

[8] Lau J., Bloch P., Schäffer L., Pettersson I., Spetzler J., Kofoed J., Madsen K., Knudsen L.B., McGuire J., Steensgaard D.B. (2015). Discovery of the Once-Weekly Glucagon-Like Peptide-1 (GLP-1) Analogue Semaglutide. J. Med. Chem..

[9] Mueckler M., Thorens B. (2013). The SLC2 (GLUT) family of membrane transporters. Mol. Aspects Med..

[10] Berger C., Zdzieblo D. (2020). Glucose transporters in pancreatic islets. Pflug. Arch. Eur. J. Physiol..

[11] Keller M.P., Choi Y., Wang P., Davis D.B., Rabaglia M.E., Oler A.T., Stapleton D.S., Argmann C., Schueler K.L., Edwards S. (2008). A gene expression network model of type 2 diabetes links cell cycle regulation in islets with diabetes susceptibility. Genome Res..

[12] Doege H., Bocianski A., Joost H.G., Schürmann A. (2000). Activity and genomic organization of human glucose transporter 9 (GLUT9), a novel member of the family of sugar-transport facilitators predominantly expressed in brain and leucocytes. Biochem. J..

[13] Lisinski I., Schurmann A., Joost H.G., Cushman S.W., Al-Hasani H. (2001). Targeting of GLUT6 (formerly GLUT9) and GLUT8 in rat adipose cells. Biochem. J..

[14] Byrne F.L., Olzomer E.M., Brink R., Hoehn K.L. (2018). Knockout of glucose transporter GLUT6 has minimal effects on whole body metabolic physiology in mice. Am. J. Physiol.-Endocrinol. Metab..

[15] Caruana B.T., Byrne F.L., Knights A.J., Quinlan K.G.R., Hoehn K.L. (2019). Characterization of Glucose Transporter 6 in Lipopolysaccharide-Induced Bone Marrow–Derived Macrophage Function. J. Immunol..

[16] Maedera S., Mizuno T., Ishiguro H., Ito T., Soga T., Kusuhara H. (2019). GLUT6 is a lysosomal transporter that is regulated by inflammatory stimuli and modulates glycolysis in macrophages. FEBS Lett..

[17] Byrne F.L., Poon I.K., Modesitt S.C., Tomsig J.L., Chow J.D., Healy M.E., Baker W.D., Atkins K.A., Lancaster J.M., Marchion D.C. (2014). Metabolic vulnerabilities in endometrial cancer. Cancer Res..

[18] Coleman D.L., Hummel K.P. (1973). The influence of genetic background on the expression of the obese (ob) gene in the mouse. Diabetologia.

[19] Gordon H.M., Majithia N., MacDonald P.E., Fox J.E.M., Sharma P.R., Byrne F.L., Hoehn K.L., Evans-Molina C., Langman L., Brayman K.L. (2017). STEAP4 expression in human islets is associated with differences in body mass index, sex, HbA1c, and inflammation. Endocrine.

[20] Cantley J., Davenport A., Vetterli L., Nemes N.J., Whitworth P.T., Boslem E., Thai L.M., Mellett N., Meikle P.J., Hoehn K.L. (2019). Disruption of beta cell acetyl-CoA carboxylase-1 in mice impairs insulin secretion and beta cell mass. Diabetologia.

[21] Ehses J.A., Perren A., Eppler E., Ribaux P., Pospisilik J.A., Maor-Cahn R., Gueripel X., Ellingsgaard H., Schneider M.K., Biollaz G. (2007). Increased number of islet-associated macrophages in type 2 diabetes. Diabetes.

[22] Steiner D.J., Kim A., Miller K., Hara M. (2010). Pancreatic islet plasticity: Interspecies comparison of islet architecture and composition. Islets.

[23] Segerstolpe Å., Palasantza A., Eliasson P., Andersson E.M., Andréasson A.C., Sun X., Picelli S., Sabirsh A., Clausen M., Bjursell M.K. (2016). Single-Cell Transcriptome Profiling of Human Pancreatic Islets in Health and Type 2 Diabetes. Cell Metab..

[24] Stancill J.S., Kasmani M.Y., Khatun A., Cui W., Corbett J.A. (2021). Single-cell RNA sequencing of mouse islets exposed to proinflammatory cytokines. Life Sci. Alliance.

[25] Negrin K.A., Roth Flach R.J., DiStefano M.T., Matevossian A., Friedline R.H., Jung D., Kim J.K., Czech M.P. (2014). IL-1 signaling in obesity-induced hepatic lipogenesis and steatosis. PLoS ONE.

[26] Shoda H., Nagafuchi Y., Tsuchida Y., Sakurai K., Sumitomo S., Fujio K., Yamamoto K. (2017). Increased serum concentrations of IL-1 beta, IL-21 and Th17 cells in overweight patients with rheumatoid arthritis. Arthritis Res. Ther..

[27] Caruana B.T., Byrne F.L. (2020). The NF-κB signalling pathway regulates GLUT6 expression in endometrial cancer. Cell Signal..

[28] Tomita T., Doull V., Pollock H.G., Krizsan D. (1992). Pancreatic islets of obese hyperglycemic mice (ob/ob). Pancreas.

[29] Parween S., Kostromina E., Nord C., Eriksson M., Lindström P., Ahlgren U. (2016). Intra-islet lesions and lobular variations in β-cell mass expansion in ob/ob mice revealed by 3D imaging of intact pancreas. Sci. Rep..

[30] Ayabe H., Ikeda S., Maruyama S., Shioyama S., Kikuchi M., Kawaguchi A., Yamada T., Ikeda T. (2013). Development of an efficient genotyping method to detect obese mutation in the mouse leptin gene for use in SPF barrier facilities. J. Vet. Med. Sci..

[31] Peng B.Y., Wang Q., Luo Y.H., He J.F., Tan T., Zhu H. (2018). A novel and quick PCR-based method to genotype mice with a leptin receptor mutation (db/db mice). Acta Pharmacol. Sin..

[32] Pfaffl M.W. (2001). A new mathematical model for relative quantification in real-time RT-PCR. Nucleic Acids Res..

[33] Grubbs F.E. (1969). Procedures for Detecting Outlying Observations in Samples. Technometrics.

