# Supplementary material for: Investigating the Expression and Function of the Glucose Transporter GLUT6 in Obesity
Source: Int J Mol Sci. 2022 Aug 29;23(17):9798. doi: 10.3390/ijms23179798 (PMC9456207; doi:10.3390/ijms23179798)
Supplement: Supplementary file 1 [file ijms-23-09798-s001.zip › ijms-1812304-supplementary.pdf]

Supplementary materials

Supplementary methods

Islet isolation

Mice were sacrificed by cervical dislocation and the abdominal area cleaned with 70% ethanol. A midline incision was performed to expose the peritoneal cavity and the common bile duct was clamped at the proximal end near the liver. The collagenase solution was prepared using Liberase T-flex (Roche, 5989132001), with final concentrations of 2.272 mg/mL collagenase and 0.075 mg/mL thermolysin in Hank's Balanced Salt Solution (HBSS, Sigma H9269). Approximately 3 mL of solution was injected at the Ampulla of Vater to perfuse the common bile duct and pancreatic duct. The perfused pancreas was quickly dissected out and placed on ice. Digestion was performed in a water bath at 37°C for 16 minutes, after which samples were placed on ice and quenched with cold HBSS(+) (HBSS containing 10% neonatal calf serum (NCS, Life Technologies 26010074) and 100 U/mL penicillin and 0.1 mg/mL streptomycin (Sigma P4333)). Following vigorous shaking, samples were filtered through 440 µm-mesh Corning Netwell inserts (Merck CLS3480) into clean 50 mL centrifuge tubes, then centrifuged at 1000 rpm and 4°C for 1 min. Samples were washed twice by removing the supernatant then vigorously resuspending the pellet in fresh HBSS(+) before centrifugation at 1000 rpm and 4°C for 1 min. After the final wash, the supernatant was removed and the pellet was resuspended in Ficoll Paque Plus (GE Healthcare GEHE17-1440-03). Then, HBSS(+) was carefully added on top to produce a layered gradient. Samples were centrifuged at 1370 xg and 4°C for 22 min, after which islets localized to the interface and were extracted by collecting both liquid layers without disturbing the pellet, then filtering through 70 µm cell strainers. Islets were washed in 70 mL HBSS(+) followed by 10 mL islet culture medium (RPMI-1640 medium (Thermofisher 11875-093) supplemented with 10% foetal bovine serum (FBS), 15 mM HEPES, and 100 U/mL penicillin and 0.1 mg/mL streptomycin). The cell strainer was then inverted over a petri dish and islets were collected by washing with islet culture medium. Any acinar contamination was minimal and picked out by hand.

In-house insulin ELISA

The in-house insulin ELISA assay was established on the premise of a "sandwich ELISA". First, a 96-well MaxiSorp plate (Thermofisher) was coated using a mouse anti-insulin antibody (Meridian Bioscience E86210M) at 1 µg/mL in phosphate-buffered saline (PBS) and incubated at 4°C overnight. The plate was washed 3 times in wash buffer (PBS with 0.1% Tween20) and blocked with 5% skim milk in PBS for 2 h at room temperature. The plate was washed again with wash buffer and 100 µL of assay buffer (137 mM NaCl, 8.16 mM Na_2HPO_4, 1.47 mM KH_2PO_4, 2.68 mM KCl, 10 mM EDTA, 1% BSA, pH 7.4) was added per well. 20 µL of sample or standard was then added to the wells. Standards were prepared using Actrapid 100 IU (Novo Nordisk), which was found to produce the same signal as equivalent concentrations of mouse insulin standard from the Crystal Chem Ultra Sensitive Mouse Insulin ELISA Kit (Crystal Chem) (Figure S1). Then, 20 µL of guinea pig anti-insulin detection antibody (Bio-Rad 5330-0104G) was added at a final concentration of 5 mg/mL. The plate was incubated overnight at 4°C. Following overnight incubation, the plate was washed and incubated in goat biotin-anti-guinea pig IgG antibody (Sigma SAB3700368) diluted 1:10 000 in assay buffer for 1 h at room temperature. After another round of washing, the plate was incubated in streptavidin-HRP (Thermofisher 434323) diluted 1:10 000 in assay buffer for a further 1 h at room temperature, then washed again and developed in TMB substrate (3,3',5,5'-tetramethylbenzidine, Merck ES022) for 30 min. The reaction was stopped with 1 M HCl and absorbance was read at 450 nm and 653 nm. The difference between the absorbance at 450 nm (signal) and the absorbance at 653 nm (background) was

used for analysis. The concentrations of the unknown samples were derived from the linear portions of the curve of corrected absorbance against standard concentrations, including a logarithmic transformation for the lower end of the samples to achieve linearity.

Western blotting

Isolated islets were washed in PBS and stored at -80°C. For protein extraction, islets were lysed in RIPA buffer (150 mM sodium chloride, 1.0% NP-40 (Sigma I8896), 0.5% sodium deoxycholate, 0.1% sodium dodecyl sulfate, 50 mM Tris, pH 8.0) containing protease inhibitors (Sigma, S8830-2TAB, Australia). Samples were put through a 29G syringe, then centrifuged at 21,130 xg for 10 min at 4°C. The supernatant was collected and protein concentration was quantified using the Pierce Bicinchoninic Acid (BCA) Protein Assay Kit according to the manufacturer's instructions (ThermoScientific, 23225).

The polyacrylamide gels used were Any kD™ Mini-Protean TGX Precast gels (Bio-Rad) or prepared in-house with 10% polyacrylamide for resolving and 4% polyacrylamide for stacking using the Bio-Rad Mini-PROTEAN system according to the manufacturer's protocol. Protein lysates were mixed with 5 × Laemmli buffer (250 mM Tris (pH 6.8), 25% RO water 10 % (w/v) SDS, 25 % (v/v) glycerol, 0.2 % w/v bromophenol blue, and 5% (v/v) β-mercaptoethanol) and heated for 5 minutes at 95°C. Protein lysates (15-40 μg) were separated by SDS-PAGE and electro-transferred to a nitrocellulose membrane. As a positive control, protein lysate from NIH-3T3 mouse fibroblasts was run concurrently. Protein transfer was confirmed using Ponceau S staining. Membranes were blocked with 5% (w/v) skim milk in TBST (Tris-buffered saline with 0.1% (v/v) Tween 20) for 1 hour at room temperature.

Proteins were detected with rabbit anti-GLUT6 (Santa Cruz, sc-134538), rabbit anti-GLUT2 (Sigma, 400061, kindly gifted by Melkam Kebede's lab at the University of Sydney), and mouse anti-14-3-3 (Santa Cruz, sc-1657), overnight at 4°C. Primary antibodies were detected with donkey anti-mouse IgG (AlexaFluor790) (Abcam ab186699) or anti-rabbit IgG (AlexaFluor680) (Abcam ab186692) and membranes were scanned on the LI-COR ODYSSEY CLx System (LI-COR, Lincoln, NE, USA). Densitometry analysis was performed using Image Studio™ (LI-COR, USA) with target signal normalized over the loading control (14-3-3).

Supplementary figures

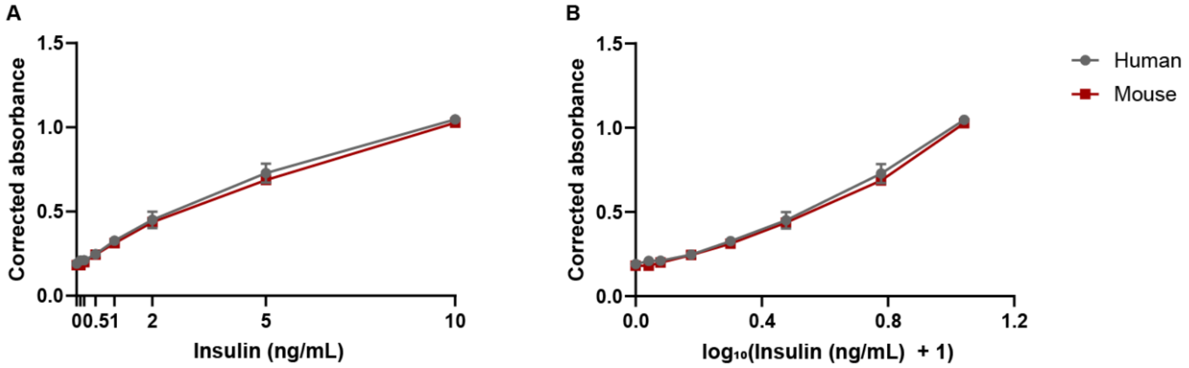

Figure S1. Comparison of standard curves produced by human and mouse insulin standards using the in-house ELISA assay. n = 3 technical replicates.

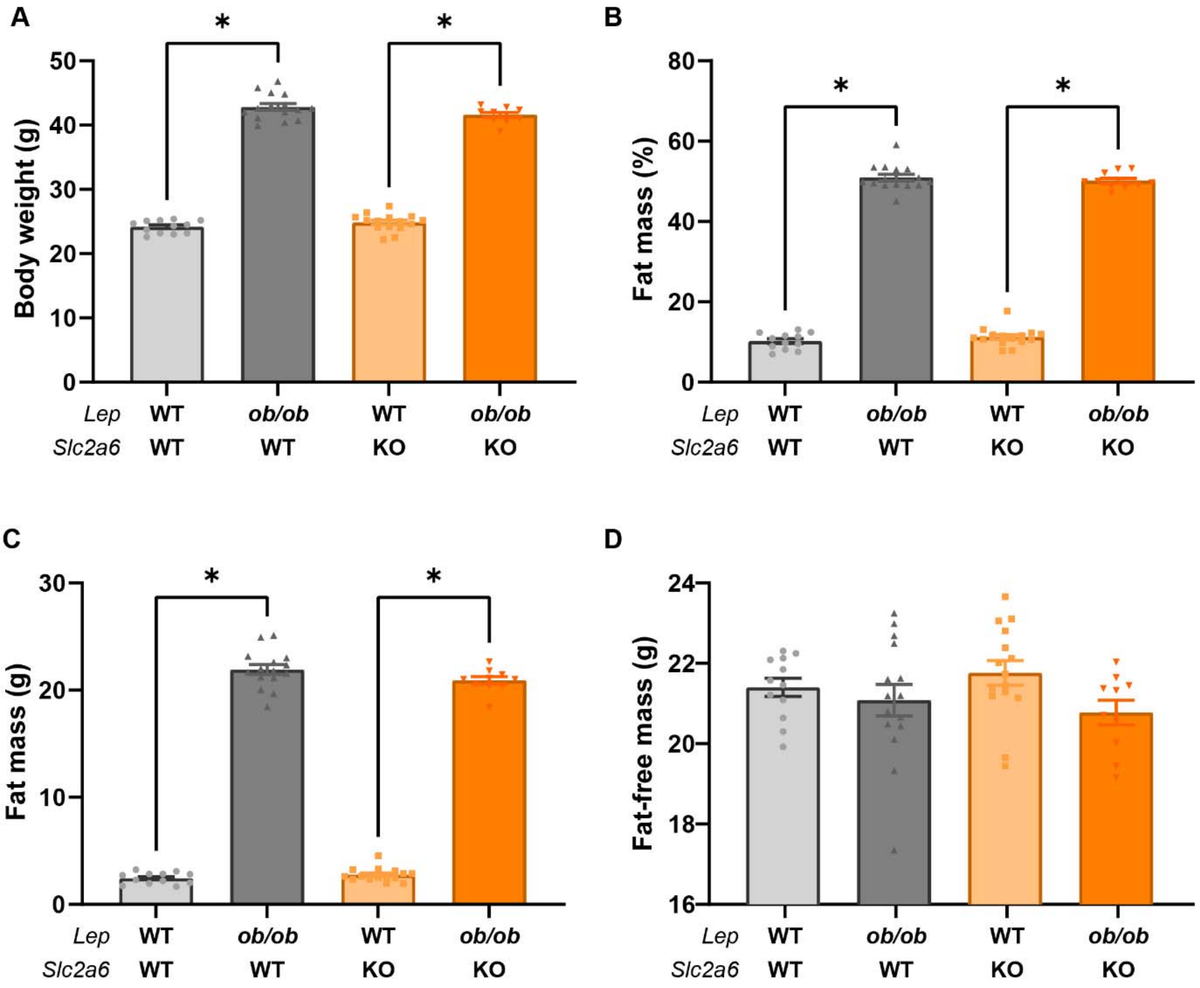

Figure S2. Body weight and composition at 10 weeks of age in male mice. Body weight (A), fat m ass as a percentage of total body mass (B), fat mass as a raw value (C) and fat-free mass (D) were measured when mice were 10 weeks of age. * indicates $p < 0.05$ by One-Way ANOVA. n = 10-15 mice per group.

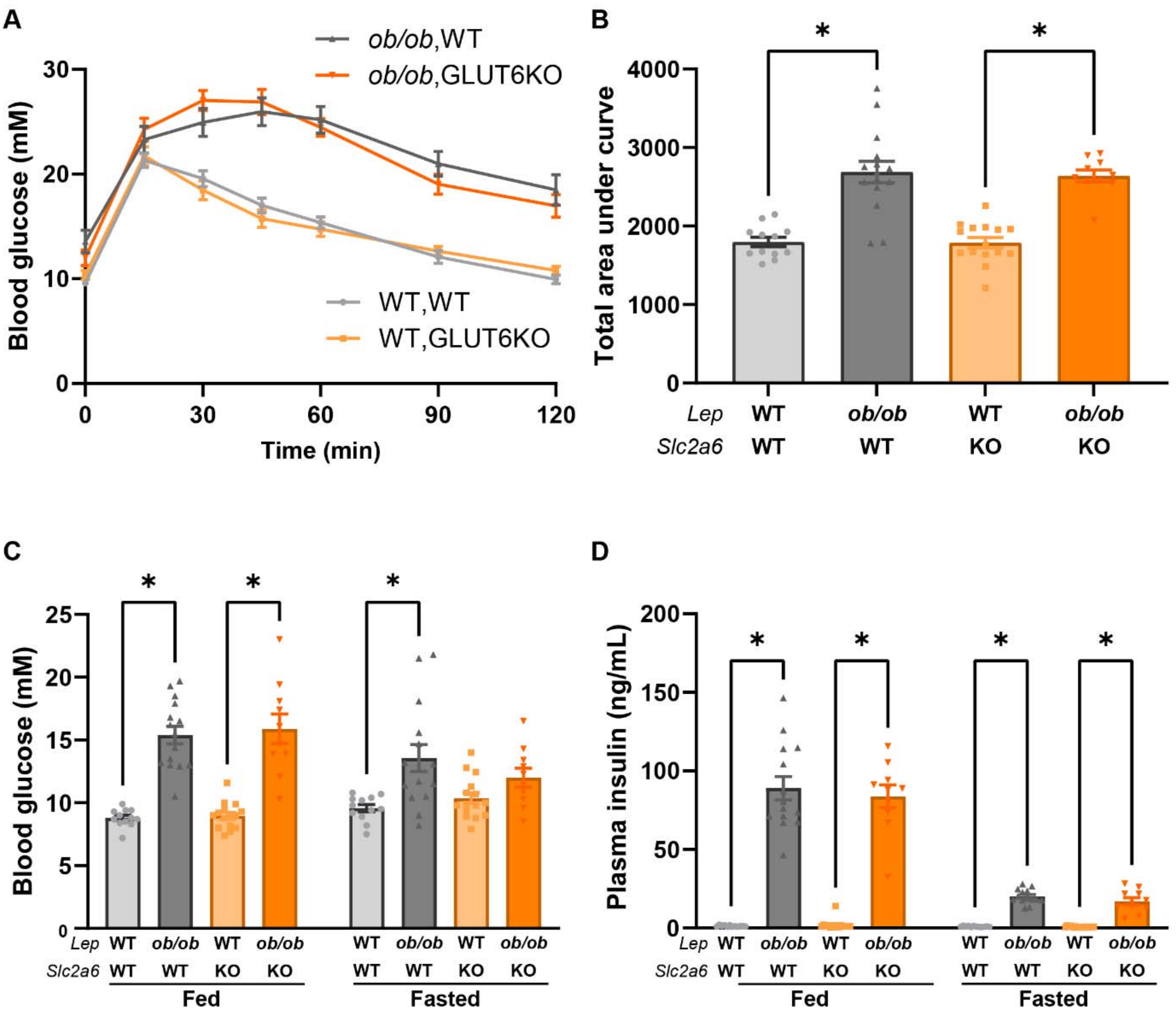

Figure S3. Glucose tolerance at 10 weeks of age in male mice. Male mice at 10 weeks of age were assessed for glucose tolerance (A-B), blood glucose (C), and plasma insulin (D). * indicates $p < 0.05$ by One-Way ANOVA. n = 10-15 mice per group.

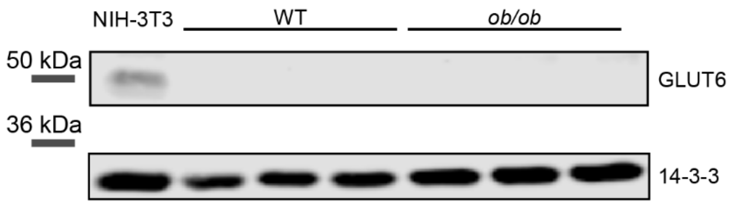

Figure S4. GLUT6 western blot of murine islet samples. Protein extracts of islets isolated from WT, WT or *ob/ob*, WT were probed for GLUT6. Mouse NIH-3T3 cell lysate was used as a positive control. n = 3 per genotype.

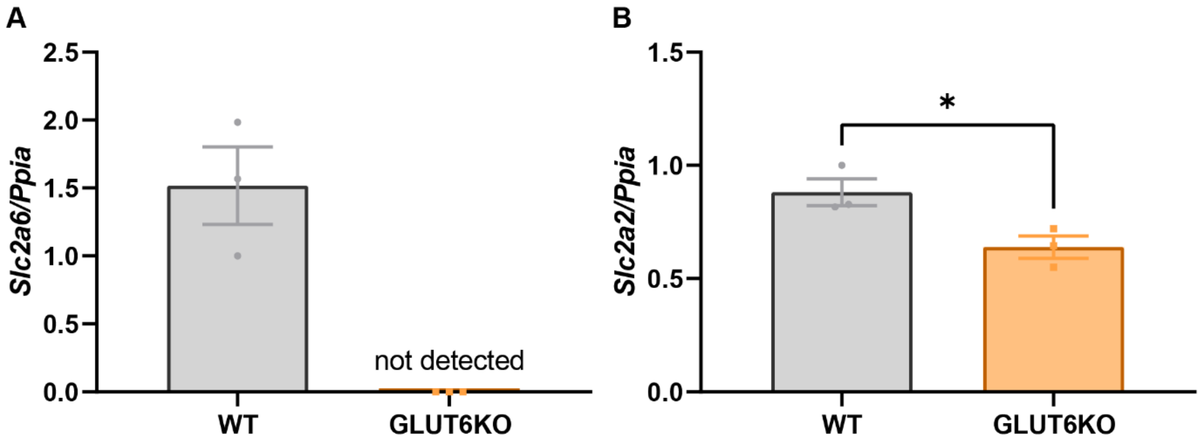

Figure S5. Glucose transporter transcript levels in isolated islets. mRNA expression of *Slc2a6* (A) and *Slc2a2* (b) in islets isolated from WT, WT and WT, GLUT6KO mice. * indicates $p < 0.05$ by Student's t-test. n = 3 per genotype.

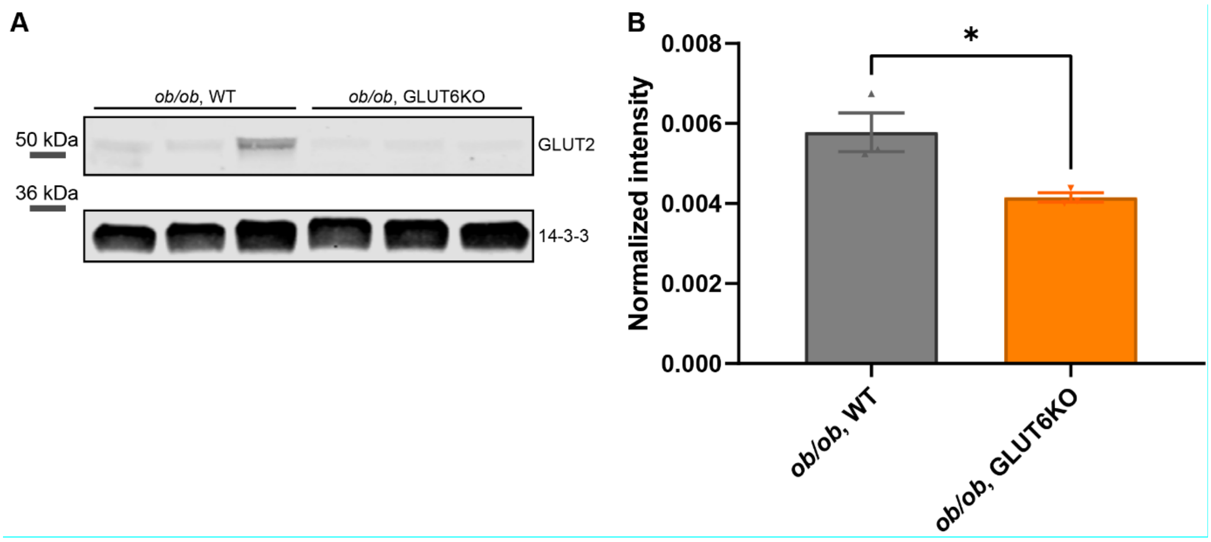

Figure S6. GLUT2 protein expression in isolated islets. Western blot of GLUT2 in *ob/ob*, WT and *ob/ob*, GLUT6KO islets (A) and quantification (normalized over 14-3-3) (B). * indicates $p < 0.05$ by Student's t-test. n = 3 per genotype.

Table S1. Donor information. Relative *SLC2A6* expression and donor information of human islet donors, as previously published [1]. "–" indicates data not collected.

Donor ID	Relative *SLC2A6* expression	Age	BMI	Sex	Time on oral hypoglycemia
Type 2 diabetic					
VP163	1.5	67	23	F	<1 year
VP168	1.6	56	28	F	0
VP093	2.4	42	29	F	–
VP173	0.6	59	24	F	0
VP141	0.1	56	29	F	–
VP143	10.7	54	53	F	0
Non-diabetic					
VP169	2.9	27	18	F	0
VP164	1.2	58	20	F	0
VP179	2.4	47	40	F	0
VP151	1	59	27	M	0
VP166	0.7	59	20	M	0
VP183	4.9	42	26	M	0

References

1. Gordon, H.M.; Majithia, N.; MacDonald, P.E.; Fox, J.E.M.; Sharma, P.R.; Byrne, F.L.; Hoehn, K.L.; Evans-Molina, C.; Langman, L.; Brayman, K.L.; et al. STEAP4 expression in human islets is associated with differences in body mass index, sex, HbA1c, and inflammation. *Endocrine* **2017**, *56*, 528-537, doi:10.1007/s12020-017-1297-2.
